# Vascular Health in American Football Players: Cardiovascular Risk Increased in Division III Players

**DOI:** 10.1155/2016/6851256

**Published:** 2016-01-24

**Authors:** Deborah L. Feairheller, Kristin R. Aichele, Joyann E. Oakman, Michael P. Neal, Christina M. Cromwell, Jessica M. Lenzo, Avery N. Perez, Naomi L. Bye, Erica L. Santaniello, Jessica A. Hill, Rachel C. Evans, Karla A. Thiele, Lauren N. Chavis, Allyson K. Getty, Tia R. Wisdo, JoAnna M. McClelland, Kathleen Sturgeon, Pam Chlad

**Affiliations:** ^1^The Hypertension and Endothelial Function with Aerobic and Resistance Training (HEART) Laboratory, Health & Exercise Physiology Department, Ursinus College, Collegeville, PA 19426, USA; ^2^Ursinus College Sports Medicine Clinic, Health & Exercise Physiology Department, Ursinus College, Collegeville, PA 19426, USA; ^3^Institute of Translational Medicine and Therapeutics, University of Pennsylvania, Philadelphia, PA 19139, USA

## Abstract

Studies report that football players have high blood pressure (BP) and increased cardiovascular risk. There are over 70,000 NCAA football players and 450 Division III schools sponsor football programs, yet limited research exists on vascular health of athletes. This study aimed to compare vascular and cardiovascular health measures between football players and nonathlete controls. Twenty-three athletes and 19 nonathletes participated. Vascular health measures included flow-mediated dilation (FMD) and carotid artery intima-media thickness (IMT). Cardiovascular measures included clinic and 24 hr BP levels, body composition, VO_2 max_, and fasting glucose/cholesterol levels. Compared to controls, football players had a worse vascular and cardiovascular profile. Football players had thicker carotid artery IMT (0.49 ± 0.06 mm versus 0.46 ± 0.07 mm) and larger brachial artery diameter during FMD (4.3 ± 0.5 mm versus 3.7 ± 0.6 mm), but no difference in percent FMD. Systolic BP was significantly higher in football players at all measurements: resting (128.2 ± 6.4 mmHg versus 122.4 ± 6.8 mmHg), submaximal exercise (150.4 ± 18.8 mmHg versus 137.3 ± 9.5 mmHg), maximal exercise (211.3 ± 25.9 mmHg versus 191.4 ± 19.2 mmHg), and 24-hour BP (124.9 ± 6.3 mmHg versus 109.8 ± 3.7 mmHg). Football players also had higher fasting glucose (91.6 ± 6.5 mg/dL versus 86.6 ± 5.8 mg/dL), lower HDL (36.5 ± 11.2 mg/dL versus 47.1 ± 14.8 mg/dL), and higher body fat percentage (29.2 ± 7.9% versus 23.2 ± 7.0%). Division III collegiate football players remain an understudied population and may be at increased cardiovascular risk.

## 1. Introduction

Hypertension and cardiovascular disease (CVD) are global health problems [1, 2]. High blood pressure (BP) has a direct relationship with increased body weight and risk of cardiac incidents and is also prevalent in professional and collegiate football players [3–5]. It is assumed that the increased physical activity the athletes perform leads to improved cardiac health, but studies report increases in CVD risk. Studies have found that Division I football players have high body fat, metabolic disease, and high resting BP levels and that BP increases over competitive seasons [6–8]. Separate research [9] has shown that Division II athletes have high BP, increased body mass index (BMI), and low high density lipoprotein (HDL) levels. There are over 70,000 NCAA football players and nearly 450 Division III schools sponsor football programs, yet research is lacking in health of Division III football players, and there is a paucity of research in vascular health of athletes overall [6–9].

Vascular health is related to CVD and can be assessed through a number of clinical modalities, including flow-mediated dilation (FMD) and carotid artery intima-media thickness (IMT) [10]. FMD is a noninvasive test, an index of NO-mediated endothelial-dependent function in humans [11, 12]. Measuring carotid artery IMT assesses vascular remodeling by quantifying thickness of the smooth muscle layer [13, 14]. Previously we have reported [15] that brachial artery FMD increased and carotid artery IMT decreased with a six-month aerobic exercise program, suggesting improvements in vascular health. However, clinical research in vascular health of athletes remains understudied. Lower FMD has been measured in professional athletes in one study, while another study found no difference in FMD between Division I football players and controls [8, 16]. Increased arterial stiffness was found [16] in Division I football players compared to controls. Other research [17] has found that professional football players have similar carotid artery IMT values to matched controls. To the best of our knowledge, no study has examined vascular health in Division III football players.

The purpose of this study was to compare vascular health between football players and controls and to examine changes in cardiovascular health over a season, providing for the first time a cardiovascular health profile in Division III football players.

## 2. Methods

### 2.1. Participants

Football players were recruited from Ursinus College NCAA Division III football team and were tested before and after season. A control group composed of nonathlete males was recruited and matched to the football players by age and by prior physical activity level (number of times reported exercise per week). The control group was tested at one point during the middle of the football season. All preseason exercise testing was completed before the football training camp, and fasting studies were completed in the first week of camp. All postseason testing was completed within two weeks of the end of the football season. Vascular studies and 24-hour Ambulatory Blood Pressure (ABP) measures were collected once during the middle of the season. Only players who completed both pre- and postseason testing were included in this analysis. For a subanalysis, players were stratified into two groups based on playing position: lineman (LM) and nonlineman (NLM).

Specific criteria for inclusion for all participants were as follows: being nondiabetic, nonsmoking, no medications that affect cardiovascular hemodynamics, no more than one antihypertensive medication, and no evidence or history of CVD, hypercholesterolemia, or renal disease. Each participant gave written informed consent and completed a health and exercise history questionnaire. The protocol was approved by the Ursinus College Institutional Review Board, and all procedures were in accordance with the ethical standards of the Helsinki Declaration.

### 2.2. Blood Pressure Measurements

Clinic BP measurements were obtained in accordance with JNC-7 guidelines [2] on three separate visits in a quiet (5 min rest), temperature controlled room, using an aneroid sphygmomanometer (Medline Industries, Mundelein, IL). For accurate readings, proper size BP cuff was used for measurements, based on the arm size of participant. BP measurements were performed in triplicate with the average of the three values used as the representative BP for that visit. The mean systolic BP and diastolic BP across the three visits are reported as the clinic BP.

Twenty-four-hour ABP monitoring was completed using a noninvasive portable BP monitor (SpaceLabs, Redmond, WA), as previously [18] described. Monitoring began in the morning of each participant's typical day. BP measures were obtained at 30 min intervals during the day and 60 min intervals at night. The following morning, each participant was asked to list their awake hours (daytime BP) and sleep hours (nighttime BP). Participant data was included in final analysis if more than 80% of the measurements were collected. Mean values were calculated for 24-hour average, for daytime, and for nighttime time-frames.

### 2.3. Glucose/Cholesterol Measurements

Fasting plasma glucose and cholesterol levels were measured using the Alere Cholestech LDX^*®*^ lipid profile system (San Diego, CA). Blood was obtained by fingerstick using a 35 *μ*L lithium heparin-coated capillary tube and tested immediately. Lipid profile cassettes were inserted into the Cholestech to analyze blood samples. Previously, fingerstick lipid profile values were correlated (*r* > 0.95) with venous plasma values measured in clinical diagnostic laboratories (Alere), and this meets the National Cholesterol Education Program criteria [19] for agreement between methods.

### 2.4. Body Composition Measurements

Body composition was measured by whole-body bioelectrical impedance (BIA) using the single frequency impedance instrument (ImpediMed DF50, San Diego, CA) following an overnight fast in a quiet, temperature controlled room. Participants were asked to refrain from salty foods, exercise, medication, alcohol, and caffeine for at least 10 hours prior to the test. Height and weight were measured using a calibrated electronic scale without shoes. BIA was measured in accordance with the manufacturer's instructions at 50 kHz on the right side of the body. Two electrodes were placed on the dorsal right hand and foot while the athletes were lying in a supine position. Three measurements were taken, and the mean values of impedance, phase, resistance, and reactance were used for calculations of total fat and fat-free mass.

### 2.5. VO_2_ Exercise Test

A maximal graded exercise test was performed to determine cardiorespiratory fitness. The Bruce protocol was performed with continuous measurement of breath-by-breath gas sampling to measure oxygen consumption (VO_2_) using a calibrated metabolic cart (TrueOne 2400, ParvoMedics, Sandy, UT). ECG was continuously monitored (Nasiff CardioCard, Central Square, NY). BP, heart rate, and perceived exertion were measured at each stage. The treadmill test was completed using termination criteria according to guidelines [20].

### 2.6. Blood Vessel Ultrasound Measurements

Brachial artery diameter measurements using flow-mediated dilation (FMD) were collected following an overnight fast in a quiet, temperature controlled room. Each participant underwent an acclimation phase (20 min) to obtain a hemodynamic steady state. Heart rate was continuously monitored using a 3-lead ECG, and BP measurements were taken in the left arm to confirm a steady state. A 5 × 84 cm automatic cuff (E-20 rapid cuff inflator; D.E. Hokanson Bellevue, WA) was placed around the right forearm distal to the olecranon process following established guidelines [21] for assessing FMD. Baseline images were obtained longitudinally 2 to 10 cm above the antecubital fossa by 2D high resolution ultrasound system, using a 5 to 12 MHz multifrequency linear array transducer. Once a satisfactory image was obtained, the right arm was secured, the position was marked, and the transducer was stabilized using a clamp. Minor corrections of transducer placement were made to maintain optimal imaging. Doppler velocity was measured via ultrasound, Doppler flow signals were corrected at an insonation angle of 60°, and measurements were performed with the sample volume placed mid-artery. Measurements were recorded for 30 seconds at baseline; then the automatic forearm cuff was then inflated to 250 mmHg and maintained for 5 minutes. Diameter and velocity recordings resumed before cuff deflation and continued for 2 minutes thereafter, while postischemia images were collected. Ultrasound FMD videos were recorded using the GE Logiq E (GE Medical Systems, Chicago, IL) and downloaded to a separate computer using Movavi Video Editor (Movavi, St Louis, MO). Arterial diameters were analyzed using the Brachial Analyzer for Research (Medical Imaging Applications, Coralville, IA). The highest 10-second interval throughout the 2-minute collection period represented the peak hyperemic diameter. Velocity and diameter measurements were converted to local shear stress using the following equation [22]: shear stress = 8 × *μ* × *V*_*H*_/*D*_BL_, where *μ* is blood viscosity, assumed to be 0.035 dyne seconds/cm^2^, *V*_*H*_ is the peak posthyperemia velocity, and *D*_BL_ represents the baseline diameter. FMD reported is the percent increase in diameter from baseline and is calculated as FMD = (peak hyperemic diameter – baseline diameter)/baseline diameter. The same operator performed all FMD measurements.

On the same day as FMD measurements, carotid artery IMT images were recorded and automatically calculated as previously [15] described. Images were obtained and measurements made using the GE Logiq E ultrasound system and automated calculation software (Auto-IMT Software Option, GE Medical Systems, Chicago, IL). Three measures were collected of the posterior wall of the common carotid artery, as per established guidelines [13]. The average of all readings was calculated, and this value is reported.

### 2.7. Statistical Analyses

Data are expressed as mean ± the standard deviation (SD). Distribution of all variables was examined using the Shapiro-Wilk test of normality. Nonparametric tests were used when appropriate. Pre- and postseason values were compared using the paired samples *t*-test or the paired samples Wilcoxon signed-rank test. Independent *t*-tests were used to compare differences between football players and controls and between LM and NLM. Pearson correlation was used to determine if there were relationships between the variables and was further examined by linear regression analysis. Statistical significance was set at *P* < 0.05. All statistical analyses were performed using SPSS version 19.0 (SPSS Inc., Chicago, IL, USA).

## 3. Results

### 3.1. Participants

Twenty-seven football players were recruited from the Ursinus College NCAA Division III football team. Due to injuries or scheduling issues, four athletes did not complete postseason testing and were excluded from the current analysis. Thus 23 athletes (12 LM and 11 NLM) were included in this analysis. Nineteen physically active noncollegiate athlete males were recruited from the college. They were matched to the football players by age (20.8 ± 2.0 yrs control, 19.8 ± 1.0 yrs football players) and by physical activity level (4.9 ± 1.5 times exercise/week control, 5.6 ± 0.9 times exercise/week football players).

### 3.2. Vascular Health and Blood Pressure

Table 1 shows the comparison of vascular and cardiovascular health measures between football players and matched controls. Football players had thicker carotid artery IMT compared to the control group (0.496 ± 0.06 mm versus 0.462 ± 0.07 mm, *P* < 0.05). Football players also had larger baseline diameter (4.3 ± 0.5 mm versus 3.7 ± 0.6 mm, *P* < 0.05) and peak brachial diameter during FMD (4.7 ± 0.6 mm versus 4.1 ± 0.7 mm, *P* < 0.05), but percent change in FMD was similar between groups (8.5 ± 4.5% versus 9.9 ± 3.3%, *P* = 0.93). Football players also had higher fasting glucose levels (91.6 ± 6.5 mg/dL versus 86.6 ± 5.8 mg/dL, *P* < 0.05), higher body fat percentage (29.2 ± 7.9% versus 23.2 ± 7.0%, *P* < 0.05), and lower fasting HDL levels (36.5 ± 11.2 mg/dL versus 47.1 ± 14.8 mg/dL, *P* < 0.05) compared to controls. All of these markers indicate a worse vascular and cardiovascular health profile in football players compared to controls.

**Figure 1:**
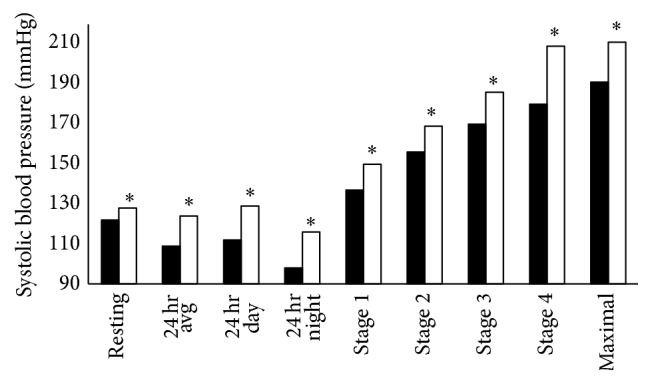
Comparison of systolic blood pressure levels between control (solid bars) and football players (open bars). Significance: ^*∗*^ *P* < 0.05 compared to control group.

Isolated systolic BP is associated with pathophysiology, is the most common form of hypertension, and is the focus of risk stratification [23]. For every measurement, systolic BP was higher in football players compared to controls.  Figure 1 shows that systolic BP was higher in football players at rest, during a full 24-hour period, at night, during all submaximal exercise stages, and at maximum exercise (all *P* < 0.05). There was no difference between the groups in any measure for diastolic BP levels.

### 3.3. Changes in Health over the Football Season

Table 2 displays changes in cardiovascular health for the football players following a collegiate season. In the entire group, only HDL increased over the season (36.5 ± 11.2 to 42.4 ± 10.8 mg/dL, *P* < 0.01). When compared by position, at both pre- and postseason testing, LM had higher body weight, higher body fat percent, lower HDL levels, and lower VO_2 max_ levels, all suggesting an inferior cardiovascular profile. After completion of the season, the LM group had an increase in HDL (32.2 ± 9.5 to 39.0 ± 9.0 mg/dL, *P* < 0.01) and triglyceride levels (107.6 ± 69 to 126.3 ± 83.2 mg/dL, *P* < 0.05), and the NLM group had a decrease in body weight (84.5 ± 4.9 to 83.2 ± 4.6 kg, *P* < 0.05) and fasting glucose levels (89.7 ± 6.0 to 86.7 ± 6.9 mg/dL, *P* < 0.05).

## 4. Conclusion

This is the first report of cardiovascular health, vascular function, and 24-hour ABP levels in a group of Division III athletes. We found thicker carotid artery IMT levels, higher systolic BP at all measurement points, higher body fat, and lower VO_2 max_ levels in football players when compared to controls. When football players were compared by position, we found that no difference exists in percent FMD or in carotid artery IMT. Finally, we confirm for the first time in Division III football players, what other studies [24, 25] have shown, LM are heavier and less fit compared to NLM.

Limited research evaluates the effect of fitness on common carotid IMT in athletes, and to the best of our knowledge no study has reported IMT measures in Division III football players. It has been shown [26] that otherwise healthy adults with lower cardiorespiratory fitness have higher IMT values, suggesting subclinical atherosclerosis. We found similar results in our study in both LM and NLM, suggesting that football players, regardless of position, may have increased risk for atherosclerosis. One study [27] reported that carotid artery IMT was higher in young professional football players compared to controls and concluded that the increased IMT in athletes may be the result of intermittent exposure to elevated arterial pressures during exercise, leading to elevated carotid wall stress. However, a separate study [17] reported no significant differences in carotid artery IMT levels between professional football players and inactive controls. The varied findings related to fitness and IMT in athletes could be related to differences in training programs; thus further studies are needed. On the other hand, the effect of BP on carotid artery IMT is established, and it is known that increased BP is related to higher common carotid IMT values [28]. We are the first to confirm this in Division III football players.

In our study, there was no difference between the groups in FMD or FMD/shear levels. Traditionally, a diminished FMD response has been associated with reduced physical activity and increased risk of CVD. Dobrosielski et al. [8] compared CVD risk factors, cardiovascular structure, and function between Division I football players (stratified by position) and controls. Dobrosielski et al. reported no significant difference in FMD between LM, NLM, and controls. We confirmed their findings in a group of Division III football players.

Research [3] reports 19.2% prevalence of hypertension and 61.9% prevalence of prehypertension in collegiate football players. According to the AHA, [29] preparticipation screening exams should identify cardiovascular risk related to physical activity. These exams include measures of heart rate, height, weight, and a clinic BP; yet, this data is not enough to protect athletes from cardiac incidents. Current recommendations [2] for accurate assessment of BP-related cardiovascular risk include 24-hour ABP as an adjunct to clinic BP measures, yet many athletic programs do not screen athletes with ABP monitoring. We are the first to report 24-hour ABP levels in a group of collegiate athletes. We found that 24-hour average, daytime, and nighttime systolic BP was higher in football players compared to the control group, which could be related to increased CVD risk. A recent study [30] in professional football players also found a reduced nighttime drop in BP in many of the players. Therefore, ABP monitoring along with clinic BP may be important. Other studies [7] have found that BP increases over a season in Division I players. When examining changes over the collegiate season, we found no change in clinic BP from pre- to postseason testing.

Future studies should further examine ABP and how clinic BP levels change in collegiate football players of all divisions. Other future studies should examine vascular health in Division III athletes to confirm our findings. Finally, given the large difference in body weight between positions, potential for metabolic syndrome should be considered and examined.

The limitations of our study are related to the participant population and sample size. Although we matched the control population to the athletes by number of exercise sessions per week, the participants may not accurately represent a comparable control for the football players, considering the type of exercise that football players undergo. The study sample size overall was small, but this was due to time restraints to get all player testing done in a set time-frame. Also, the sample was derived from one small Division III college, which may not be representative of all football athletes. These limitations emphasize the need for a further, larger study that assesses surrogate markers of vascular health and cardiovascular risk along with other cardiovascular outcomes in collegiate athletes.

In conclusion, collegiate football players may be at increased risk for impaired cardiovascular or vascular health, and Division III athletes are a large collegiate population of athletes who remain understudied. This is the first report of vascular function, cardiovascular health, and 24-hour ABP levels in a group of Division III football players. Compared to active controls, football players have thicker carotid artery IMT levels, higher systolic BP at all measurement points, higher body fat, and lower VO_2 max_ levels in football players when compared to controls. Also, we confirm for the first time in Division III football players, what other studies have shown in professional football players and collegiate Division I players, LM are heavier and less fit compared to fitness matched controls.

## Figures and Tables

**Table 1:** Comparison between football players and control.

Variable	Football players	Control
Age	19.8 ± 1.0	20.8 ± 2.0
Height (cm)	178.8 ± 4.6	178.0 ± 6.1
Body weight (kg)	103.8 ± 20.6	78.7 ± 10.6
Body fat (%)	29.2 ± 7.9	23.2 ± 7.0^*∗*^
SBP (mmHg)	128.2 ± 6.4	122.4 ± 6.8^*∗*^
DBP (mmHg)	74.8 ± 4.1	73.9 ± 6.3
Glucose (mg/dL)	91.6 ± 6.5	86.6 ± 5.8^*∗*^
Total cholesterol (mg/dL)	136.6 ± 23.9	157.1 ± 36.8
HDL (mg/dL)	36.5 ± 11.2	47.1 ± 14.8^*∗*^
Triglycerides (mg/dL)	98.2 ± 55.2	102.1 ± 60.5
LDL (mg/dL)	83.2 ± 18.2	97.3 ± 33.9
VO_2max⁡_ (mL/kg/min)	42.4 ± 8.4	49.6 ± 7.8^*∗*^
Baseline BA diameter (mm)	4.3 ± 0.5	3.7 ± 0.6^*∗*^
FMD (%)	8.5 ± 4.5	9.9 ± 3.3
FMD/shear	0.61 ± 0.3	0.60 ± 0.3
IMT (mm)	0.496 ± 0.06	0.462 ± 0.07^*∗*^

Data are presented as mean ± SD. ^*∗*^Post hoc significantly different from control, *P* < 0.05. SBP, systolic blood pressure; DBP, diastolic blood pressure; HDL, high density lipoprotein; LDL, low density lipoprotein; VO_2max⁡_, oxygen consumption; BA, brachial artery; FMD, flow-mediated dilation; IMT, intima-media thickness.

**Table 2:** Football player responses over a season.

	Nonlineman		Lineman	
	Preseason	Postseason	Preseason	Postseason
Age	19.9 ± 1.1	—	19.8 ± 0.9	—
# Times exercise/week	5.8 ± 1.0	—	5.6 ± 0.9	—
# Games played	6.4 ± 3.0	—	4.9 ± 4.2	—
Height (cm)	176.0 ± 4.1	—	180.8 ± 4.2	—
Body weight (kg)	84.5 ± 4.9	83.2 ± 4.6^*∗*^	117.0 ± 14.1^*∗∗*^	117.1 ± 14.7^*∗∗*^
Clinic SBP (mmHg)	130.2 ± 11.6	133.0 ± 6.6	129.0 ± 6.8	132.4 ± 7.8
Clinic DBP (mmHg)	75.1 ± 4.9	74.3 ± 7.5	76.4 ± 3.9	74.1 ± 4.9
Glucose (mg/dL)	89.7 ± 6.0	86.7 ± 6.9^*∗*^	93.7 ± 6.5	93.3 ± 9.0
Total cholesterol (mg/dL)	137.0 ± 29.0	141.4 ± 24.8	133.9 ± 16.7	142.3 ± 22.2
HDL (mg/dL)	42.0 ± 11.1	46.7 ± 11.2	32.2 ± 9.5^*∗∗*^	39.0 ± 9.0^*∗*^
Triglycerides (mg/dL)	89.3 ± 31.8	93.0 ± 23.6	107.6 ± 69.0	126.3 ± 83.2^*∗*^
LDL (mg/dL)	83.6 ± 18.1	91.6 ± 13.3	83.7 ± 15.8	82.1 ± 15.8
Body fat (%)	23.2 ± 2.7	25.9 ± 5.0	34.2 ± 7.3^*∗∗*^	31.1 ± 6.7^*∗∗*^
VO_2max⁡_ (mL/kg/min)	48.9 ± 5.2	49.0 ± 4.4	37.1 ± 6.5^*∗∗*^	38.3 ± 6.5^*∗∗*^

Data are mean ± SD. ^*∗*^ *P* < 0.05 from preseason to postseason.  ^*∗∗*^ *P* < 0.05 between groups. SBP, systolic blood pressure; DBP, diastolic blood pressure; HDL, high density lipoprotein; LDL, low density lipoprotein; VO_2_, volume of oxygen consumption.
